# Bimetallic Zinc-Iron-Modified Sugarcane Bagasse Biochar for Simultaneous Adsorption of Arsenic and Oxytetracycline from Wastewater

**DOI:** 10.3390/molecules30030572

**Published:** 2025-01-27

**Authors:** Nhat-Thien Nguyen, An-Bang Lin, Chang-Tang Chang, Gui-Bing Hong

**Affiliations:** 1 Department of Chemical Engineering and Biotechnology, National Taipei University of Technology, No. 1, Sec. 3, Zhongxiao E. Rd., Taipei City 106, Taiwan; nguyennhatthien333@gmail.com; 2 Department of Environmental Engineering, National Ilan University, Yilan City 26047, Taiwan; ampang1419@gmail.com

**Keywords:** sugar cane bagasse, biochar, adsorption, arsenic, oxytetracycline

## Abstract

Arsenic (As), a highly toxic and carcinogenic heavy metal, poses significant risks to soil and water quality, while oxytetracycline (OTC), a widely used antibiotic, contributes to environmental pollution due to excessive human usage. Addressing the coexistence of multiple pollutants in the environment, this study investigates the simultaneous adsorption of As(III) and OTC using a novel bimetallic zinc-iron-modified biochar (1Zn-1Fe-1SBC). The developed adsorbent demonstrates enhanced recovery, improved adsorption efficiency, and cost-effective operation. Characterization results revealed a high carbon-to-hydrogen ratio (C/H) and a specific surface area of 1137 m^2^ g^−1^ for 1Zn-1Fe-1SBC. Isotherm modeling indicated maximum adsorption capacities of 34.7 mg g^−1^ for As(III) and 172.4 mg g^−1^ for OTC. Thermodynamic analysis confirmed that the adsorption processes for both pollutants were spontaneous (ΔG < 0), endothermic (ΔH > 0), and driven by chemical adsorption (ΔH > 80 kJ mol^−1^), with increased system disorder (ΔS > 0). The adsorption mechanisms involved multiple interactions, including pore filling, hydrogen bonding, electrostatic attraction, complexation, and π-π interactions. These findings underscore the potential of 1Zn-1Fe-1SBC as a promising adsorbent for the remediation of wastewater containing coexisting pollutants.

## 1. Introduction

Arsenic (As) is a highly toxic and carcinogenic heavy metal that poses a significant threat to global soil and water quality. Its contamination has been widely reported in river systems across various countries, including the United States, China, Turkey, and Vietnam. The presence of arsenic in water is associated with severe environmental and health risks, leading to diseases such as black foot disease, diabetes, hyperkeratosis, cancer, hypertension, and neuropathy. Regulatory agencies have established stringent standards to mitigate these risks; for instance, the Ministry of Environment of the Executive Yuan has set arsenic limits in groundwater at 0.05 mg L^−1^ and 0.005 mg L^−1^ for general and drinking water protection zones, respectively. Despite these efforts, arsenic pollution continues to be a pressing issue worldwide [1]. Arsenic primarily exists in aqueous solutions as arsenite (As(III)) and arsenate (As(V)) [2]. Generally, inorganic arsenic compounds exhibit higher toxicity compared to their organic counterparts. Among the inorganic species, As(III) is more toxic than As(V), as it binds with greater affinity to vicinal sulfhydryl groups, interacting with various proteins and inhibiting their function. Furthermore, As(III) demonstrates greater stability than As(V), attributed to its electronic configuration [3,4].

Oxytetracycline (OTC), a widely used antibiotic in human medicine and animal husbandry, has become another critical environmental pollutant. While antibiotics are invaluable in combating bacterial infections and improving life expectancy, their extensive use and improper disposal have resulted in significant contamination. Incomplete metabolic degradation of antibiotics in humans (70–90%) exacerbates this issue, as unmetabolized residues are released into the environment. According to the World Health Organization, antibiotic pollution poses one of the greatest threats to global health, food security, and ecological stability, with adverse impacts on plants, animals, and ecosystems [5].

Pharmaceutical and personal care products (PPCPs) have been linked to abnormalities in human and microbial systems, as reported in several studies [6,7,8]. These compounds, which contaminate rivers and other aquatic ecosystems, have become a significant focus of research due to their adverse effects on aquatic organisms. Efforts to eliminate PPCPs from the environment have been extensively studied [9,10]. PPCPs have been detected in effluents [11], surface waters such as rivers [12], and within sewage treatment systems. Among the wide range of pharmaceuticals and over-the-counter products, antimicrobials are widely used in both human medicine and animal husbandry [13]. Their antimicrobial properties make them resistant to degradation within biological systems [14], necessitating the development of advanced adsorption techniques for the effective removal of oxytetracycline (OTC).

A variety of technologies have been developed to address heavy metal and antibiotic contamination in water, including chemical precipitation [15], biological treatment [16], ion exchange [17], filtration [18], flotation [19], redox reactions [20], electrochemistry [21], adsorption [22], and membrane filtration [23]. Among these, adsorption has emerged as a promising method due to its effectiveness in removing and recovering pollutants. However, traditional adsorbents often suffer from high costs, limited recovery efficiency, and technical challenges, hindering their widespread adoption. Sugarcane bagasse (SCB) biochar, a carbon-rich material derived from agricultural waste, represents a sustainable and cost-effective alternative for environmental remediation. However, unmodified biochar typically exhibits low adsorption efficiency, necessitating chemical or structural modifications to enhance its pollutant removal capabilities. SCB biochar has shown promise as an adsorbent for heavy metal removal from soil and water, attributed to its microporous structure and large specific surface area [24,25]. Its surface is enriched with functional groups such as carboxyl (-COOH), hydroxyl (-OH), and amino (-NH_2_), facilitating adsorption via mechanisms like electrostatic attraction, cation exchange, surface complexation, and electron donation [26]. A noteworthy example is the development of a zinc-iron bimetallic system for water purification applications. Chen et al. [27] prepared modified walnut shell biochar (ZF@WBC) through zinc-iron bimetallic oxide modification at 600 °C under oxygen-limited conditions. Characterization revealed that ZF@WBC exhibited a significantly enhanced surface area (376 m^2^ g^−1^) and total pore volume (0.205 cm^3^ g^−1^) compared to unmodified walnut shell biochar. Adsorption experiments demonstrated that ZF@WBC achieved a maximum adsorption capacity of 104.26 mg g^−1^ for Pb(II), which is 2.57 times higher than that of unmodified biochar, highlighting its improved performance [27].

This study focuses on the development of a chemically modified biochar derived from SCB, an abundant agricultural byproduct. By incorporating zinc chloride (ZnCl_2_) and ferric chloride (FeCl₃) during the modification process, the biochar’s adsorption properties, including surface area, pore structure, and functional groups, are significantly improved. This modified biochar not only demonstrates enhanced adsorption performance for As(III) and OTC but also offers advantages such as reduced operational costs, material recyclability, and reusability. The primary objectives of this research are to develop a cost-effective biochar capable of simultaneously removing As(III) and OTC, optimize the composition of zinc and iron in the modification process, establish optimal adsorption parameters, and elucidate the underlying mechanisms driving pollutant removal. This work aims to provide an innovative and sustainable solution for addressing the challenges of multi-pollutant contamination in water systems.

## 2. Results and Discussion

### 2.1. Characterization Analysis

#### 2.1.1. SEM Analysis Results

**Figure 1:**
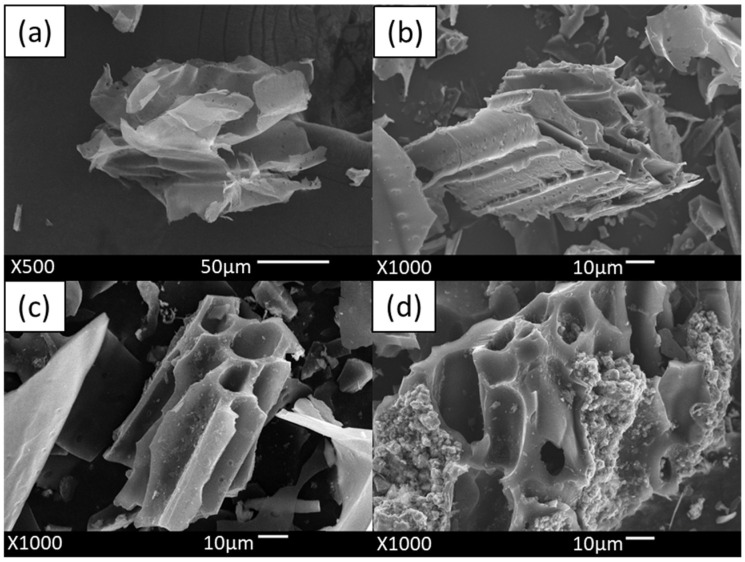
SEM analysis results: (**a**) SCB, (**b**) SBC, (**c**) 1Zn-1Fe-1SBC, and (**d**) 1Zn-1Fe-1SBC-As-OTC.

Scanning electron microscopy (SEM) was employed to investigate the surface morphology and properties of SCB, SBC, 1Zn-1Fe-1SBC, and 1Zn-1Fe-1SBC-As-OTC (after adsorption of As and OTC onto 1Zn-1Fe-1SBC). The SEM images are presented in Figure 1. The analysis revealed that the biochar materials, including SCB, exhibited pores of varying sizes and uneven, multilayered surface structures. Figure 1b shows that SBC possesses a relatively well-developed pore structure compared to SCB (Figure 1a), primarily due to the decomposition of organic matter on the SCB surface under high-temperature pyrolysis, which resulted in the formation of a stable pore network on the SBC surface. In Figure 1c, the surface of 1Zn-1Fe-1SBC modified with ZnCl_2_ and FeCl_3_ is shown to exhibit a significantly enhanced pore structure and particle formation, attributed to ZnCl_2_ impregnation. Consistent with findings by Lin et al. [28], the SCB pores underwent substantial corrosion following FeCl_3_ modification, leading to an increased specific surface area and the deposition of metal residues on the material’s surface. The modifications using ZnCl_2_ and FeCl_3_ enhanced the material’s specific surface area and pore volume. BET and EDS analyses further confirmed this enhancement, showing an increase in specific surface area from 16.8 m^2^ g^−1^ to 1136.8 m^2^ g^−1^ and pore volume from 0.02 cm^3^ g^−1^ to 0.55 cm^3^ g^−1^ as detailed in Table 1. Additionally, EDS mapping revealed the uniform distribution of Zn and Fe elements across the material’s surface, as illustrated in Figure S1 in the Supplementary Materials. These modifications using ZnCl_2_ and FeCl_3_ effectively improved the material’s adsorption properties by significantly enhancing its surface area and pore volume.

Figure 1d illustrates the surface morphology of the 1Zn-1Fe-1SBC-As-OTC material, which shows numerous particles deposited on the surface, forming deposition-induced pores. These particles correspond to adsorbed As(III) and OTC, leading to a slight reduction in specific surface area (from 1136.8 m^2^ g^−1^ to 945.7 m^2^ g^−1^) and pore volume (from 0.55 cm^3^ g^−1^ to 0.43 cm^3^ g^−1^), as confirmed by BET (Table 1) and EDS analyses. Additionally, EDS mapping identified the presence of As on the material’s surface (as shown in Figure S2), aligning with the findings reported by Liu et al. [29]. Overall, the 1Zn-1Fe-1SBC material developed in this study successfully adsorbed As(III) and OTC, demonstrating its potential for effective pollutant removal.

#### 2.1.2. EDS Analysis Results

Energy-dispersive X-ray spectroscopy (EDS) was employed to analyze the composition and distribution of chemical elements on the surfaces of SCB, SBC, 1Zn-1Fe-1SBC, and 1Zn-1Fe-1SBC-As-OTC materials. The results, presented in Table 2, reveal the primary elemental compositions of the materials. For SCB and SBC, the main elements detected were C, O, and Si, while the dominant elements for 1Zn-1Fe-1SBC and 1Zn-1Fe-1SBC-As-OTC included C, O, Si, Zn, Fe, and As. As shown in Figure S3, the distribution of C and O on the surface of SCB was uniform, attributed to the abundance of oxygen-containing functional groups and surface-bound water molecules [30]. The presence of Si on the SCB surface likely results from the interaction between sugarcane straw and soil, incorporating nutrients and minerals from the soil into the sugarcane stalks [31]. Figure S4 illustrates that the O content in SBC decreases significantly, while the levels of C and Si increase, as confirmed by the numerical elemental compositions provided in Table 2. This change is attributed to the substantial reduction in organic matter during the high-temperature pyrolysis of SCB, leading to an increase in the relative proportions of inorganic C and Si. As confirmed in Figure S1, the EDS analysis demonstrates the successful incorporation of Zn and Fe onto the surface of 1Zn-1Fe-1SBC following modification with ZnCl_2_ and FeCl_3_. These elements are observed to be widely and uniformly distributed across the material’s surface. Notably, no detectable Cl was found after retesting the EDS, further confirming the successful dechlorination of ZnCl_2_ and FeCl_3_ during the modification process. This evidence supports the conclusion of “complete dechlorination” and aligns with the data presented in Table 2. Figure S2 illustrates that after the adsorption of As(III) and OTC by 1Zn-1Fe-1SBC, the presence of As and OTC on the material’s surface is evident. Additionally, the EDS results confirm the presence of N, indicating the successful adsorption of OTC on the material surface. The O content increases significantly during the adsorption process, which can be attributed to the specific interaction mechanisms between As(III) and the biochar (SBC) surface. Specifically, the metal oxyhydroxide nanoparticles present on the SBC surface facilitate the replacement of OH-ligands in As(III) molecules, forming mono- and bidentate complexes. These complexes enable As(III) to adhere to the surface, contributing to the observed increase in O content. These findings underscore the material’s efficacy in capturing target pollutants through surface adsorption and validate the compositional integrity and adsorption mechanisms discussed in this study.

#### 2.1.3. FTIR Analysis Results

**Figure 2:**
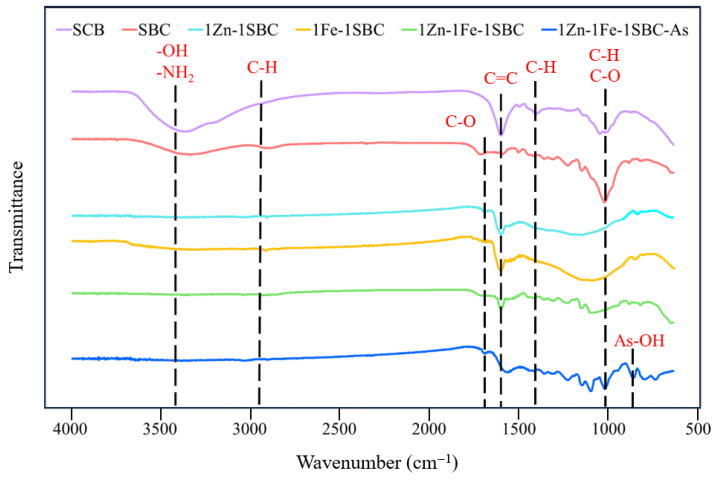
FTIR analysis of various biochars.

Fourier-transform infrared (FTIR) spectroscopy was employed to investigate the functional groups present in the materials (SCB, SBC, 1Zn-1Fe-1SBC, and 1Zn-1Fe-1SBC-As) within the range of 650–4000 cm^−1^. The results, presented in Figure 2, reveal that SCB, due to its complex organic and inorganic composition, exhibits a variety of functional groups and carbohydrate-related features. The characteristic peaks observed in the range of 3000–3600 cm^−1^ correspond to the stretching vibrations of -OH and -NH_2_ groups, which are associated with water molecules and amine groups, respectively. The peaks observed at 2849 and 2930 cm^−1^ are attributed to the C-H stretching vibrations in the -CH and -CH_2_ groups. All SBC samples exhibit C=O vibrations at 1760 cm^−1^, which is consistent with previous studies on biochar and its derivatives [32,33]. The stretching vibration of the aromatic C=C ring corresponds to the band observed at 1500–1600 cm^−1^. The peak band at 1470–1500 cm^−1^ corresponds to bending vibrations that may include contributions from C–H, CH_2_, and CH_3_ groups, as distinguishing these vibrations in this range can be challenging without additional analysis [34]. Vibrations of C–C bonds are observed in the 1000–1100 cm^−1^ range. However, in the spectra of (bio)polymers, this range typically overlaps with contributions from C–O and C–H bonds, which complicates precise assignments [35]. Notably, after adsorption of As(III) onto 1Zn-1Fe-1SBC, a new band appears at 852 cm^−1^, corresponding to As-OH bending vibrations. This observation aligns with findings reported by Chen et al. [36], Rago et al. [37], Coates et al. [38], and Park et al. [39]. For SBC, 1Zn-1SBC, 1Fe-1SBC, and 1Zn-1Fe-1SBC, the bending vibrations of -OH and -NH_2_ groups were significantly reduced, leaving prominent peaks for C=O and C-O groups. This is attributed to the decomposition of water molecules and amine groups during high-temperature pyrolysis. According to Minaei et al. [40], the modification and pyrolysis of biomass (e.g., sludge) result in a simplified FTIR spectrum, with distinct C=O and C-O bending vibrations observed at 1560 and 1016 cm^−1^, respectively. Consistent with the findings of Park et al. [39] and Cen et al. [41], the bending vibration of As-OH appears in the 800–900 cm^−1^ range after As adsorption. In this study, the high-temperature pyrolysis of SCB led to substantial sintering of organic matter in SBC, 1Zn-1SBC, 1Fe-1SBC, and 1Zn-1Fe-1SBC, resulting in the disappearance of most carbon-containing functional groups. However, the adsorption of As(III) by 1Zn-1Fe-1SBC introduced an As-OH bending vibration band at 852 cm^−1^, indicative of successful adsorption.

#### 2.1.4. TGA Analysis Results

**Figure 3:**
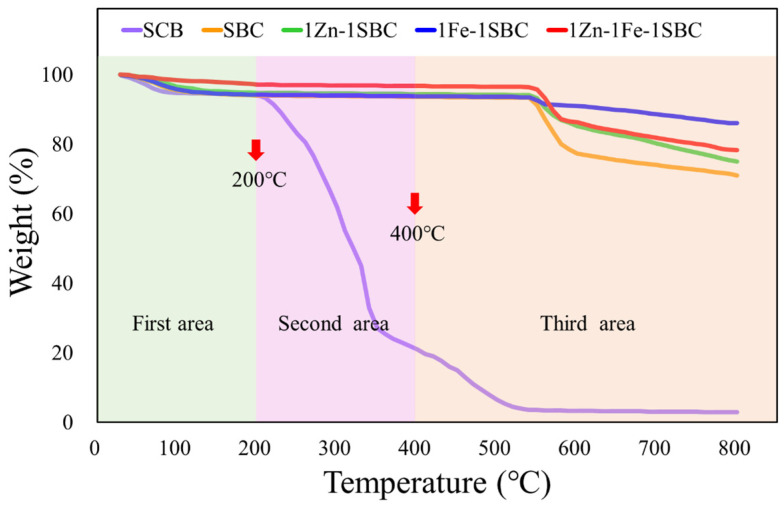
TGA analysis of various biochars.

Thermogravimetric analysis (TGA) was employed to evaluate the thermal stability and compositional characteristics of the materials SCB, SBC, 1Zn-1SBC, 1Fe-1SBC, and 1Zn-1Fe-1SBC. The materials were subjected to a temperature range of 25–800 °C under a nitrogen atmosphere. The weight loss profiles are illustrated in Figure 3. The initial weight loss, attributed to water evaporation and the volatilization of organic compounds, was approximately 6.0%, 5.9%, 5.2%, 5.8%, and 29% for SCB, SBC, 1Zn-1SBC, 1Fe-1SBC, and 1Zn-1Fe-1SBC, respectively [42]. The second stage of weight loss occurred in the temperature range of 200–400 °C, associated with the thermal decomposition of lignin, hemicellulose, and cellulose. During this stage, SCB showed a weight loss of approximately 73.1%, while SBC, 1Zn-1SBC, 1Fe-1SBC, and 1Zn-1Fe-1SBC exhibited minimal weight losses of 0.5%, 0.4%, 0.3%, and 0.4%, respectively. The third stage of weight loss, observed between 400–800 °C, was primarily due to the decomposition of residual organic matter. The weight losses during this phase were 18.1%, 22.6%, 19.4%, 7.8%, and 18.4% for SCB, SBC, 1Zn-1SBC, 1Fe-1SBC, and 1Zn-1Fe-1SBC, respectively. The reduced organic content in Zn- and Fe-modified biochars (1Zn-1SBC, 1Fe-1SBC, and 1Zn-1Fe-1SBC) contributed to their lower overall weight losses. Moreover, the presence of Zn and Fe, which are inorganic elements with high melting points, enhanced the thermal stability of the materials at elevated temperatures.

These observations are consistent with previous studies. According to Marx et al. [43], Stylianou et al. [44], and Kaikiti et al. [45], weight loss between 30–200 °C is predominantly associated with water evaporation and organic volatilization. The rapid weight loss between 200–1000 °C is attributed to the high-temperature decomposition of lignin (250–900 °C), cellulose (300–400 °C), and hemicellulose (220–315 °C). Hu et al. [46] reported similar trends in thermogravimetric behavior for biochars and metal-doped biochars. For example, undoped biochar (BC) exhibited a weight loss of 88% between 300–600 °C, while Zn-Fe-modified biochar (ZF-BC) and tri-metallic spinel biochar (MZF-BC) showed reduced weight losses of 62% and 50.7%, respectively. This reduction is attributed to the lower organic matter content in metal-doped biochars. Consistently, the Zn- and Fe-modified biochar 1Zn-1Fe-1SBC in this study demonstrated superior thermal stability compared to other biochars, particularly at high temperatures.

#### 2.1.5. BET Analysis Results

**Figure 4:**
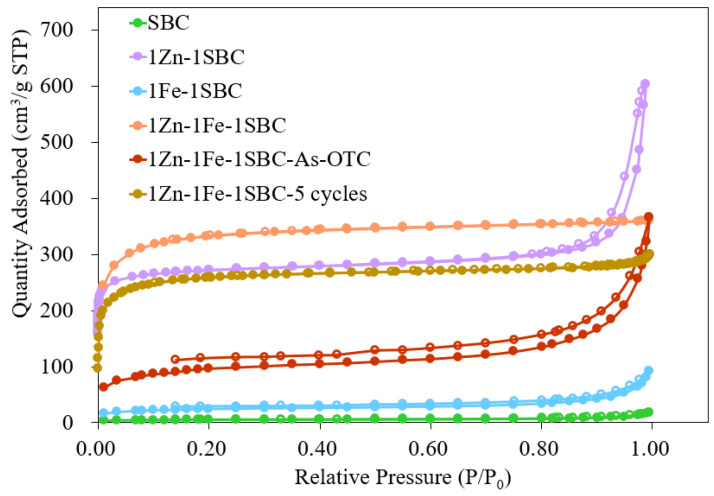
The N_2_ adsorption–desorption isotherm of biochars.

**Figure 5:**
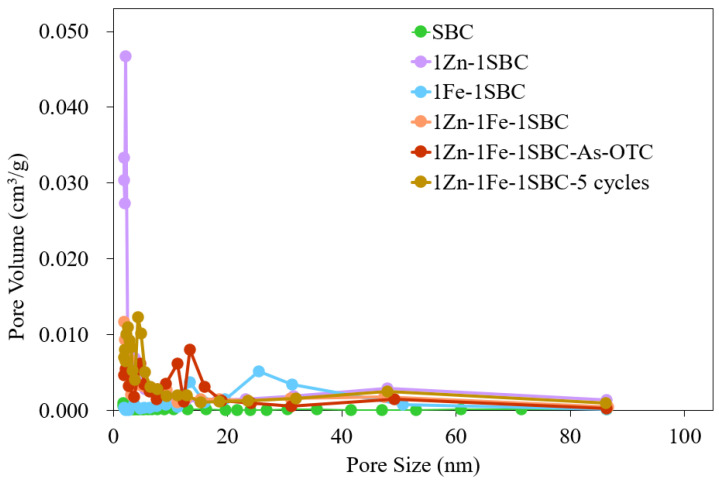
The pore size distribution patterns of biochars.

The pore characteristics, specific surface area, and pore size distribution of SBC, 1Zn-1SBC, 1Fe-1SBC, 1Zn-1Fe-1SBC, and 1Zn-1Fe-1SBC-As-OTC were analyzed using Brunauer–Emmett–Teller (BET) isothermal adsorption/desorption methods. The results, presented in Figure 4 and Figure 5 and summarized in Table 1, provide insights into the influence of material modifications and adsorption processes on these parameters. The specific surface areas of SBC, 1Zn-1SBC, 1Fe-1SBC, 1Zn-1Fe-1SBC, and 1Zn-1Fe-1SBC-As-OTC were 16.8, 1314.5, 97.3, 1136.8, and 945.7 m^2^ g^−1^, respectively. Correspondingly, their pore volumes were 0.02, 0.64, 0.31, 0.55, and 0.43 cm^3^ g^−1^, while their average pore sizes were 4.65, 1.92, 3.28, 1.98, and 3.05 nm. These results demonstrate that modifications with ZnCl_2_ and FeCl_3_ significantly increased both the specific surface area and pore volume compared to unmodified SBC. The enhancement in these parameters is primarily attributed to the etching effects of ZnCl_2_ and FeCl_3_, which created new cavities and pores on the material surface. For instance, the specific surface area of SBC increased from 16.8 to 1136.8 m^2^ g^−1^, and the pore volume increased from 0.02 to 0.55 cm^3^ g^−1^ after modification. However, after the adsorption of As(III) and OTC onto 1Zn-1Fe-1SBC, both specific surface area and pore volume decreased significantly, with the specific surface area reducing from 1136.8 to 945.7 m^2^ g^−1^ and the pore volume reducing from 0.55 to 0.43 cm^3^ g^−1^. This reduction is attributed to pore blockage caused by the adsorption of As(III) and OTC. Furthermore, after five adsorption cycles (1Zn-1Fe-1SBC5 cycles), the specific surface area and pore volume of 1Zn-1Fe-1SBC decreased slightly, from 1136.8 to 989.5 m^2^ g^−1^ and from 0.55 to 0.45 cm^3^ g^−1^, respectively, indicating that the pore structure remained intact despite repeated usage.

These findings are consistent with results reported by Li et al. [47], where biochar modified with ZnSO₄ and FeCl_3_ exhibited a significant increase in specific surface area and pore volume compared to unmodified biochar. For example, the specific surface area of Mikania micrantha Kunth biochar increased from 4.2 m^2^ g^−1^ to 54.1 m^2^ g^−1^ after modification, with a corresponding increase in pore volume from 0.02 to 0.14 cm^3^ g^−1^. However, after the adsorption of OTC, these parameters decreased, suggesting effective adsorption and pore occupation. In this study, the modification of SBC with ZnCl_2_ and FeCl_3_ significantly enhanced the specific surface area and pore volume, making it a highly effective material for the adsorption of As(III) and OTC. The observed decrease in these parameters post-adsorption confirms the successful loading of these contaminants onto the modified material.

#### 2.1.6. XPS Analysis Results

**Figure 6:**
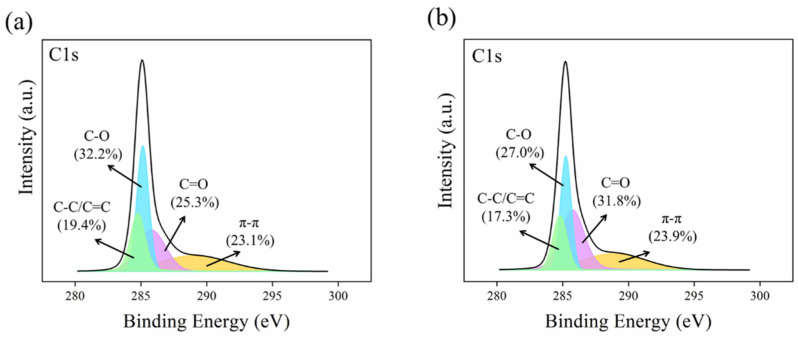
Electron spectra of C1s from XPS: (**a**) 1Zn-1Fe-SBC and (**b**) 1Zn-1Fe-1SBC-As-OTC.

To investigate the composition, content, and molecular structure characteristics of the carbon (C) element on the surfaces of 1Zn-1Fe-1SBC and 1Zn-1Fe-1SBC-As-OTC, X-ray photoelectron spectroscopy (XPS) analysis was conducted on the C1s electron spectra. As shown in Figure 6, the binding energies of C-C/C=C, C-O, C=O, and π-π* shakeup satellite interactions were detected at 284.8, 285.1, 285.8, and ~6.8 eV, respectively [48]. From Figure 6a, the binding energies corresponding to C-C/C=C, C-O, C=O, and π-π* shakeup satellite features were observed on the 1Zn-1Fe-1SBC surface, while Figure 6b reveals that these binding energies were also present on the 1Zn-1Fe-1SBC-As-OTC surface, with respective area ratios of 17.3%, 27.0%, 31.8%, and 23.9%. The increased π-π* shakeup satellite area (23.9%) in 1Zn-1Fe-1SBC-As-OTC compared to 1Zn-1Fe-1SBC suggests enhanced π-π electron interactions between the biochar (acceptor) and the adsorbed As(III) and OTC molecules (donors). This phenomenon is consistent with the findings of Zhang et al. [49], who reported an increase in the π-π* shakeup satellite area from 8.90% to 9.32% in Sycamore Flocs Biochar after the adsorption of OTC-HCl, attributed to the interaction of π-π electron donor-acceptor systems.

**Figure 7:**
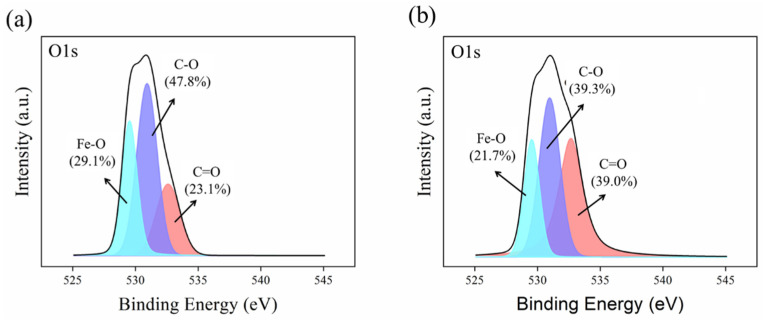
O1s electron spectra of XPS: (**a**) 1Zn-1Fe-SBC and (**b**) 1Zn-1Fe-1SBC-As-OTC.

To further analyze the oxygen (O) element on the surfaces of 1Zn-1Fe-1SBC and 1Zn-1Fe-1SBC-As-OTC, XPS O1s spectra were examined. Figure 7 indicates the presence of Fe-O, C-O, and C=O binding energies on the biochar surface, located at 529.5, 530.9, and 532.6 eV, respectively. From Figure 7a, the binding energy areas for Fe-O, C-O, and C=O on the 1Zn-1Fe-1SBC surface were 29.1%, 47.8%, and 23.1%, respectively. In contrast, Figure 7b shows that the respective binding energy areas on the 1Zn-1Fe-1SBC-As-OTC surface were reduced to 21.7%, 39.3%, and 39.0%. The C-O and C=O binding energy variations observed in Figure 7b can indeed be attributed to the sorbed OTC, as OTC contains its own C-C, C=C, C-O, and C=O functional groups. These functional groups may contribute to the changes in the C-O and C=O regions. However, we believe that the reduction in the Fe-O binding energy area is primarily due to interactions between the Fe sites and the sorbed OTC molecules. These interactions likely cause a shift in the electron distribution, resulting in changes in the Fe-O contribution, which indicates that the adsorption of OTC has influenced the surface structure of the material. The results of Yoon et al. [50] strongly suggested adsorption of As occurred via a specific chemical reaction between As and Fe–O functional groups on magnetite. The overall results suggest the use of FeCl_3_ is a feasible practical approach to control the intrinsic pH of biochar and impart additional functionality that enables effective treatment of As.

**Figure 8:**
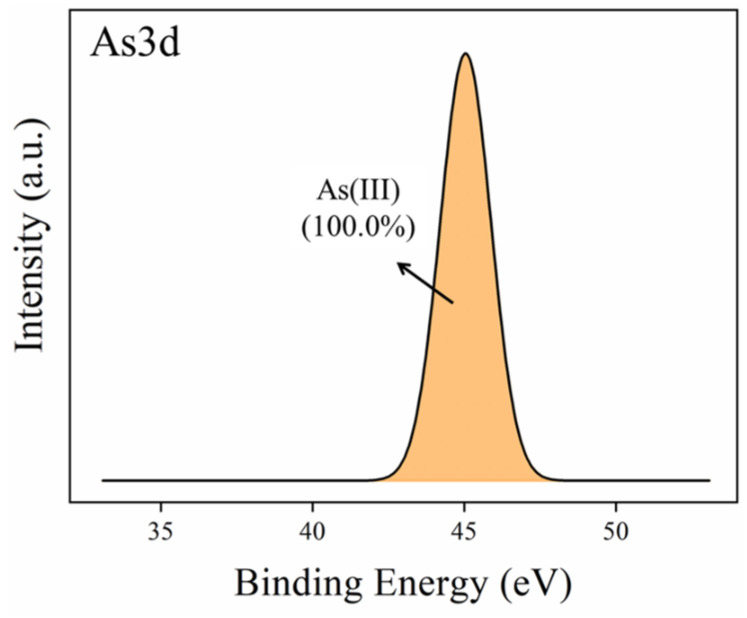
As3d electron spectra of XPS from 1Zn-1Fe-SBC-As-OTC.

To explore the presence of arsenic (As) on the surface of 1Zn-1Fe-1SBC-As-OTC, the As3d spectra were analyzed via XPS. As shown in Figure 8, the binding energy of As(III) was identified at 45.0 eV, with an area ratio of 100%, confirming the successful adsorption of As(III) onto the surface of 1Zn-1Fe-1SBC. This observation is consistent with the findings of Zama et al. [51], who reported the appearance of As(III) binding energy at 45.1 eV on the surface of Aspen Wood Biochar after the adsorption of As(III). These results collectively demonstrate the successful adsorption of As(III) and OTC onto the surface of the modified biochar, elucidating the molecular interactions responsible for these adsorption processes.

### 2.2. Results of Simultaneous Adsorption of As(III) and OTC

#### 2.2.1. Comparison of Different Biochars 

**Figure 9:**
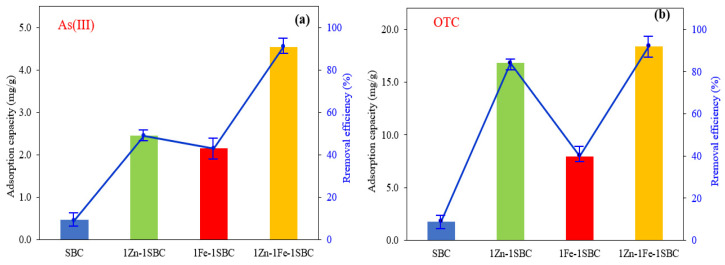
Comparison of biochar absorption performance: (**a**) As(III) and (**b**) OTC. (C_0_ of As(III) = 1 ppm, C_0_ of OTC = 4 ppm, dose = 0.2 g/L, T = 25 °C, pH = 5).

The adsorption efficiencies of four biochars—SBC, 1Zn-1SBC, 1Fe-1SBC, and 1Zn-1Fe-1SBC—towards As(III) were 9%, 49%, 43%, and 91%, respectively, corresponding to adsorption capacities of 0.06, 2.46, 2.15, and 4.55 mg g^−1^, as illustrated in Figure 9. The adsorption capacities observed in this study are higher than those reported by Lin et al. [52] for metal-biochar (3.6 mg g^−1^). Similarly, the adsorption efficiencies of these biochars for OTC were 10%, 84%, 40%, and 92%, respectively, with adsorption capacities of 0.25, 16.8, 7.98, and 18.4 mg g^−1^. The results indicate that 1Zn-1Fe-1SBC exhibited the highest adsorption efficiencies for both As(III) and OTC. This superior performance can be attributed to the synergistic effects of ZnCl_2_ and FeCl_3_ during the modification process, which enhanced the specific surface area, pore volume, and functional group availability on the biochar surface. These structural and chemical improvements significantly contributed to the adsorption of both As(III) and OTC.

#### 2.2.2. Effect of Fe and Zn Content

**Figure 10:**
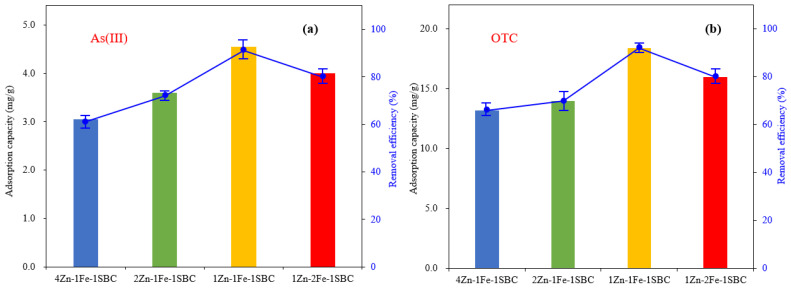
Comparison of biochar absorption performance with non-identical Zn and Fe content: (**a**) As(III) and (**b**) OTC. (C_0_ of As(III) = 1 ppm, C_0_ of OTC = 4 ppm, dose = 0.2 g L^−1^, T = 25 °C, pH = 5).

The adsorption efficiencies of biochars modified with varying ratios of ZnCl_2_ and FeCl_3_—4Zn-1Fe-1SBC, 2Zn-1Fe-1SBC, 1Zn-1Fe-1SBC, and 1Zn-2Fe-1SBC—for As(III) were 61%, 72%, 91%, and 80%, respectively, with corresponding adsorption capacities of 3.05, 3.60, 4.55, and 4.00 mg g^−1^. Similarly, the adsorption efficiencies for OTC were 66%, 70%, 92%, and 80%, respectively, with adsorption capacities of 13.2, 14.1, 18.4, and 16.0 mg g^−1^, as illustrated in Figure 10. These results demonstrate that increasing the proportion of metal modifiers initially enhances the adsorption efficiency of biochar for both As(III) and OTC. However, when the metal content exceeds an optimal level, the adsorption efficiency declines. This reduction is primarily attributed to excessive metal content, which can block pores on the biochar surface, thereby reducing the available adsorption sites. Comparable findings were reported by Li et al. [53], who investigated the effect of ZnCl_2_ content on the adsorption efficiency of chromium using domestic sewage sludge carbon (DSSC). The adsorption capacities for DSSC mixed with 33%, 50%, 60%, and 67% ZnCl_2_ were 66.1, 68.8, 66.3, and 55.9 mg g^−1^, respectively. The study revealed that while an optimal ZnCl_2_ ratio enhances porosity and adsorption capacity, excessive ZnCl_2_ leads to pore blockage, reducing adsorption efficiency. Similarly, the optimal adsorption efficiency for As(III) and OTC in this study was achieved with an appropriate Zn-to-Fe ratio in the biochar. Both insufficient and excessive metal content adversely impacted adsorption performance. This underscores the importance of balancing the metal modifier ratios to maximize the adsorption capabilities of the biochar.

#### 2.2.3. Effect of pH

This study investigates the impact of SBC, 1Zn-1SBC, 1Fe-1SBC, and 1Zn-1Fe-1SBC materials on the adsorption capacity for As(III) and OTC at different pH values (3, 5, 7, 9, and 11). The initial concentrations of As(III) and OTC were 1 ppm and 4 ppm, respectively. The material dosage was 0.2 g L^−1^, the temperature was maintained at 25 °C, and the contact time was set to 120 min to evaluate the effect of pH on the adsorption of As(III) and OTC.

As shown in Figure S5, 1Zn-1Fe-1SBC exhibited the highest adsorption efficiency for As(III), with removal rates of 89%, 91%, 86%, 82%, and 76% at pH values of 3, 5, 7, 9, and 11, respectively. The corresponding adsorption capacities were 4.43, 4.56, 4.32, 4.08, and 3.77 mg g^−1^. The isoelectric points of SBC, 1Zn-1SBC, 1Fe-1SBC, and 1Zn-1Fe-1SBC were found to be at pH 3.9, 5.2, 6.5, and 5.9, respectively (Figure S6). When the pH is lower than the isoelectric point of the biochar, the materials carry a positive charge, while at pH values above the isoelectric point, the materials are negatively charged. The experimental results indicate that the adsorption of As(III) is most effective when the pH is between 3 and 5. As the pH increases from 5 to 11, the adsorption capacity for As(III) decreases. This decrease is primarily due to the pH’s influence on the biochar, which not only affects the adsorption reaction but also alters the chemical form of As(III) in the solution. As(III) has three dissociation constants: pK_a1_, pK_a2_, and pK_a3_, with values of 9.2, 12.1, and 13.4, respectively. Between pH 3.22 and 7.46, As(III) predominantly exists as neutral ions (H_2_AsO_3_), and between pH 7.46 and 14.0, it exists mainly in anionic forms (H_2_AsO_3_^−^, HAsO_3_^2−^, and AsO_3_^3−^).

Similarly, Figure S7 shows that 1Zn-1Fe-1SBC exhibits the best adsorption efficiency for OTC, with removal rates of 84%, 92%, 84%, 79%, and 72% at pH values of 3, 5, 7, 9, and 11, respectively. The corresponding adsorption capacities were 16.9, 18.4, 16.8, 15.8, and 14.4 mg g^−1^. The experimental results demonstrate that the adsorption capacity for OTC increases as the pH value rises from 3 to 5, but decreases as the pH increases further from 5 to 11. OTC has three dissociation constants: pK_a1_, pK_a2_, and pK_a3_, with values of 3.22, 7.46, and 8.94, respectively. Between pH 3.22 and 7.46, OTC primarily exists in its cationic (OTC^+^) and neutral (OTC^±^) forms. Between pH 7.46 and 8.94, OTC is mainly in its anionic form (OTC^−^), and between pH 8.94 and 14.0, it exists predominantly in the forms OTC^−^ and OTC^2−^.

### 2.3. Isothermal Adsorption Model Correlation Results

The adsorption behavior of 1Zn-1Fe-1SBC for As(III) and OTC was evaluated using the Langmuir, Freundlich, and Temkin isothermal adsorption models, which are among the most widely applied models for adsorption studies. The fitting results are summarized in Table 3 and Figure S8. The correlation coefficients (R^2^) for the Langmuir model fitting of 1Zn-1Fe-1SBC adsorption of As(III) and OTC were both 0.999, indicating excellent agreement. For the Freundlich model, the R^2^ values were 0.996 and 0.997, respectively, while the Temkin model yielded R^2^ values of 0.929 and 0.926 for As(III) and OTC, respectively. These results suggest that the Langmuir model provides the best fit for describing the adsorption behavior of 1Zn-1Fe-1SBC, as its correlation coefficients are superior to those of the Freundlich and Temkin models. Based on the Langmuir model analysis, the maximum adsorption capacities for 1Zn-1Fe-1SBC were determined to be 34.72 mg g^−1^ for As(III) and 172.41 mg g^−1^ for OTC. According to the Freundlich model analysis, the *n*-values for As(III) and OTC adsorption were both greater than one, indicating that the adsorption process is favorable. The Temkin model analysis showed that higher K_T_ values correspond to lower adsorption capacities, with the K_T_ values indicating a stronger adsorption effect of 1Zn-1Fe-1SBC for OTC compared to As(III).

For comparison, Xia et al. [54] reported a maximum adsorption capacity of 27.67 mg g^−1^ for ZnCl_2_-activated biochar, as determined by the Langmuir model. Furthermore, Zhao et al. [55] emphasized that the n-value from the Freundlich model is a critical indicator of adsorption efficiency, with n > 1 signifying effective adsorption of pollutants, such as antibiotics, by biochar. In conclusion, the 1Zn-1Fe-1SBC synthesized in this study exhibits superior adsorption capacities for As(III) and OTC, outperforming previously reported materials and demonstrating its potential as an effective adsorbent.

### 2.4. Adsorption Kinetic Model Correlation Results

The adsorption kinetics of 1Zn-1Fe-1SBC for As(III) and OTC were analyzed using the pseudo-first-order and pseudo-second-order kinetic models, which are widely applied to study adsorption processes. The fitting results are presented in Table 4. The squared correlation coefficients (R^2^) for the pseudo-first-order model for the adsorption of As(III) and OTC ranged from 0.845 to 0.994 and 0.894 to 0.992, respectively. For the pseudo-second-order model, the R^2^ values ranged from 0.968 to 0.989 for As(III) and from 0.970 to 0.994 for OTC. These results indicate that both kinetic models are suitable for describing the adsorption behavior of 1Zn-1Fe-1SBC, as the R^2^ values for both models are generally greater than 0.9. The findings are consistent with prior research. Norberto et al. [56] analyzed the adsorption kinetics of As(III) and As(V) using Canola Straw Biochar (CSB). Their results showed that the R^2^ value for the pseudo-first-order model was 0.962 for As(III) and 0.983 for As(V), while the R^2^ value for the pseudo-second-order model was 0.997 for As(III) and 0.958 for As(V). These findings suggest that both physical and chemical adsorption processes are involved in the adsorption of As(III) and As(V) by CSB, as both models provided good fits. Similarly, the results of this study demonstrate that the adsorption kinetics of As(III) and OTC onto 1Zn-1Fe-1SBC can be effectively described by both the pseudo-first-order and pseudo-second-order models, indicating the occurrence of both physical and chemical adsorption mechanisms.

The adsorption process of As(III) and OTC onto 1Zn-1Fe-1SBC was also analyzed using the intra-particle diffusion model to investigate the internal diffusion mechanisms. The initial concentrations of As(III) and OTC were set at 1.0 ppm and 4.0 ppm, respectively, with a material dosage of 0.2 g L^−1^. The experiments were conducted at 25 °C, with the pH adjusted to 5, and the contact time ranging from 0 to 120 min. The adsorption reaction curves for both As(III) and OTC were divided into three distinct stages, representing different diffusion mechanisms. The first stage corresponds to volumetric diffusion (K_1_), the second stage to thin-film diffusion (K_2_), and the third stage to intra-particle diffusion (K_3_). The regression results, as summarized in Table 5, indicate that the K_1_ values for As(III) and OTC adsorption were 0.922 mg/g·min^0.5^ and 3.012 mg/g·min^0.5^, respectively. The K_2_ values were 0.411 mg/g·min^0.5^ and 1.390 mg/g·min^0.5^, while the K_3_ values were 0.116 mg/g·min^0.5^ and 0.246 mg/g·min^0.5^, respectively. The analysis shows that the K_1_ values in the first stage are significantly higher than the K_2_ and K_3_ values, indicating that volumetric diffusion is the dominant adsorption mechanism. Thin-film diffusion and intra-particle diffusion are secondary processes that contribute to the overall adsorption mechanism. Similar findings were reported by Feng et al. [57], who studied the adsorption of As(III) using iron-modified biochar (Fe-BC300 and Fe-BC500). Their adsorption reaction curves were also divided into three stages. In their study, the reaction rate constants for the first stage (K_1_, 0–60 min) were 4.79 mg/g·min^0.5^ and 3.12 mg/g·min^0.5^, respectively, which were notably higher than those of the subsequent stages. This indicates that the primary adsorption process was the diffusion of As(III) from the liquid phase to the material’s surface. The second stage (K_2_, 120–360 min) demonstrated reaction rate constants of 0.83 mg/g·min^0.5^ and 0.93 mg/g·min^0.5^, signifying the diffusion of As(III) from the surface to the inner layers of the material. In the third stage (K_3_, 960–1440 min), the reaction rate constants were 0.32 mg/g·min^0.5^ and 0.68 mg/g·min^0.5^, respectively, indicating diffusion into the pores of the material. As the adsorption capacity increased, diffusion resistance also rose, leading to a decrease in the adsorption rate until equilibrium was achieved. The results of this study suggest that the adsorption of As(III) and OTC onto 1Zn-1Fe-1SBC is primarily governed by volumetric diffusion, with thin-film diffusion playing a secondary role.

### 2.5. Thermodynamic Analysis Results

The thermodynamic parameters, including Gibbs free energy (ΔG), enthalpy (ΔH), and entropy (ΔS), for the adsorption of As(III) and OTC onto 1Zn-1Fe-1SBC were evaluated at varying temperatures. Experiments were conducted at 15, 25, 35, and 45 °C (288, 298, 308, and 318 K), respectively. The initial concentrations of As(III) and OTC were fixed at 1 ppm and 4 ppm, respectively, with a material dosage of 0.2 g L^−1^. The pH was maintained at 5, and the contact time was set to 120 min to facilitate the determination of ΔG, ΔH, and ΔS. The thermodynamic analysis results are summarized in Table 6. The negative values of ΔG for both As(III) and OTC adsorption (as shown in Table 5) indicate that the process is spontaneous. Furthermore, the magnitude of ΔG decreases significantly with increasing temperature, suggesting that higher temperatures enhance the adsorption reaction, thereby facilitating the diffusion of As(III) and OTC to the material’s surface. Notably, the ΔG values for OTC adsorption are smaller than those for As(III) adsorption, demonstrating that 1Zn-1Fe-1SBC exhibits a stronger adsorption affinity for OTC. The positive ΔH values indicate that the adsorption of As(III) and OTC is endothermic in nature. For adsorption processes where ΔH is less than 80 kJ mol^−1^, the mechanism is considered physical adsorption, whereas ΔH values exceeding 80 kJ mol^−1^ suggest chemical adsorption. In this study, the ΔH values confirm that the adsorption of As(III) and OTC by 1Zn-1Fe-1SBC involves chemical adsorption [58]. Additionally, the positive ΔS values imply an increase in system disorder during adsorption. This indicates an increased probability of collision between the adsorbent and As(III) or OTC molecules. Collectively, the ΔG, ΔH, and ΔS values reveal that the adsorption reaction is thermodynamically favorable and becomes more efficient with rising temperature.

### 2.6. Arrhenius Model Analysis Results

The reaction rate constants (k_1_) for the adsorption of As(III) and OTC by 1Zn-1Fe-1SBC at 15, 25, 35, and 45 °C (288, 298, 308, and 318 K) were analyzed using the Arrhenius model to evaluate the activation energy (E_a_) of the adsorption process at different temperatures. The results of the analysis are presented in Table 7. The calculated E_a_ values for the adsorption of As(III) and OTC by 1Zn-1Fe-1SBC were 21.82 and 26.07 kJ mol^−1^, respectively. The pre-exponential factors (A) were determined to be 32.9 and 754.3 s^−1^, respectively. According to the Arrhenius model simulation, the E_a_ value greater than 21 kJ mol^−1^ confirms that the adsorption process is governed by a chemical reaction mechanism. Furthermore, the higher E_a_ value for OTC adsorption compared to As(III) adsorption indicates that the adsorption of OTC involves a higher activation energy, suggesting a more energy-intensive reaction for OTC adsorption on 1Zn-1Fe-1SBC [59]. Jin et al. [60] reported similar findings using nanosized Fe/Ni-functionalized ZIF-8 (ZIF-8-Fe/Ni) for the adsorption of OTC. Their study, employing the Arrhenius model, found an E_a_ value of 22.9 kJ mol^−1^ with an R^2^ of 0.993. This aligns with the criterion that an E_a_ value exceeding 21 kJ mol^−1^ characterizes the adsorption process as a chemical reaction. These results further corroborate the chemical nature of the adsorption mechanism for 1Zn-1Fe-1SBC.

### 2.7. Adsorption Mechanism of 1Zn-1Fe-1SBC for As(III) and OTC

**Figure 11:**
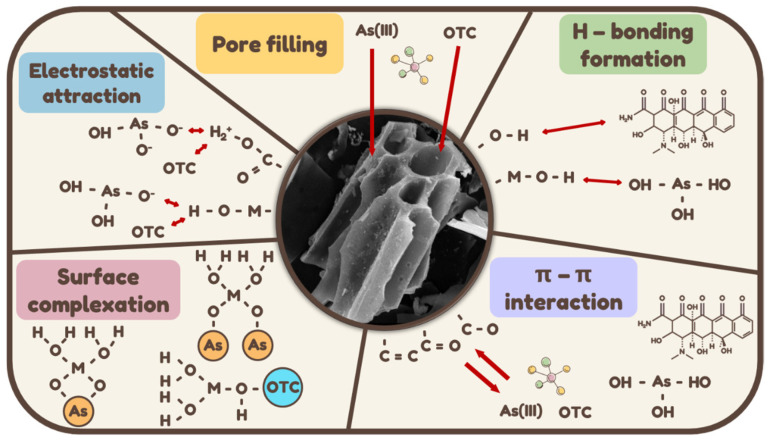
Adsorption mechanism of As(III) and OTC on 1Zn-1Fe-1SBC.

The adsorption mechanisms of 1Zn-1Fe-1SBC for As(III) and OTC were investigated in this study. A comprehensive understanding of the adsorption pathways was achieved through a combination of literature references and experimental data. Figure 11 illustrates the five primary reaction pathways involved in the adsorption process.

#### 2.7.1. Pore Blocking and Surface Effects

Energy-dispersive X-ray spectroscopy (EDS) revealed the presence of As on the surface of 1Zn-1Fe-1SBC following adsorption, while X-ray photoelectron spectroscopy (XPS) confirmed the appearance of As(III) binding energy peaks. Brunauer–Emmett–Teller (BET) analysis indicated that the specific surface area and pore volume of 1Zn-1Fe-1SBC decreased significantly after adsorption, from 1136.8 m^2^ g^−1^ and 0.55 cm^3^ g^−1^ to 945.7 m^2^ g^−1^ and 0.43 cm^3^ g^−1^, respectively. This reduction demonstrates that the adsorption of As(III) and OTC caused pore blockage. Intra-particle diffusion model analysis further confirmed that As(III) and OTC ions were successfully adsorbed into the material’s pores during the adsorption process.

#### 2.7.2. Formation of Hydrogen Bonds

The functional groups present in biochar, particularly those containing hydrogen atoms, facilitated the formation of hydrogen bonds with As(III) and OTC ions in aqueous solutions, enhancing the stability of the adsorption process [28]. Fourier-transform infrared (FTIR) spectroscopy showed an absorption band at 852 cm^−1^, corresponding to the bending vibration of As-OH after adsorption [39]. Furthermore, the high adsorption efficiency observed at pH 5 can be attributed to surface protonation, which promotes hydrogen bond formation between functional groups on 1Zn-1Fe-1SBC and the adsorbed ions [47]. These hydrogen bonds provide additional attractive forces, stabilizing the adsorption of arsenic ions.

#### 2.7.3. Electrostatic Interactions

FTIR analysis revealed that 1Zn-1Fe-1SBC possessed functional groups with varying charge states, enabling electrostatic interactions with As(III) and OTC ions in solution [28]. The adsorption behavior was strongly influenced by the pH of the solution, which determined the charge states of both the adsorbate and adsorbent. The isoelectric point of 1Zn-1Fe-1SBC was found to be pH 5.9. Under acidic conditions, As(III) primarily exists as H_3_AsO_3_ and H_2_AsO_3_^−^, reducing electrostatic repulsion. OTC exists as OTC^+^ and OTC^±^, where the latter is less repelled by the material. These electrostatic interactions played a critical role in the effective adsorption of As(III) and OTC ions at varying pH levels [61].

#### 2.7.4. Surface Complexation

FTIR spectra indicated the presence of As-OH bending vibrations at 852 cm^−1^ after adsorption, while XPS analysis demonstrated the binding of As to the surface of 1Zn-1Fe-1SBC. The functional groups and metal elements (C-O, C=O, Fe, and Zn) on the material contributed to the formation of surface complexes with As(III) and OTC ions, further enhancing the adsorption process [62].

#### 2.7.5. π-π Interactions

FTIR and XPS analyses identified functional groups such as -OH, C=C, and C=O on the surface of 1Zn-1Fe-1SBC. These groups acted as electron donors, facilitating π-π interactions that enhanced the adsorption of As(III) and OTC. XPS data for C1s electron spectroscopy showed a decrease in functional groups (C-C and C=C) and an increase in π-π interactions from 23.1% to 23.9% after adsorption, providing further evidence of the role of π-π interactions in the adsorption process [63]. In conclusion, the adsorption of As(III) and OTC by 1Zn-1Fe-1SBC involves multiple mechanisms, including pore blocking, hydrogen bond formation, electrostatic interactions, surface complexation, and π-π interactions. These synergistic effects contribute to the material’s high adsorption efficiency.

## 3. Materials and Methods

### 3.1. Preparation of Biochar

The sugarcane bagasse (SCB) was initially washed three times with ultrapure water to remove impurities and then dried at 105 °C for 24 h under ambient conditions. The dried material was pulverized using a crusher to obtain powdered pretreated SCB. The SCB was then placed in a quartz tube, and pyrolysis was performed in a tube furnace. The heating rate was set at 5 °C min^−1^, and the pyrolysis temperature was maintained at 550 °C for 2 h under an oxygen-free environment using nitrogen gas (N_2_). After the furnace cooled, the pyrolyzed product was removed and treated with 3 M HCl for 30 min at approximately 90 °C to eliminate residual organic matter. The biochar was then thoroughly washed with ultrapure water until a neutral pH (7) was achieved. The product was dried in an oven at 105 °C for 12 h and subsequently sieved through a 100-mesh sieve to obtain carbonized biochar (SBC).

### 3.2. Preparation of Modified Biochar

#### 3.2.1. Monometallic Modified Biochar

Monometallic modified biochar was synthesized by incorporating ZnCl_2_ (Shimakyu Co., Ltd., Osaka, Japan, 98%) or FeCl_3_ (Shimakyu Co., Ltd., Japan, 45%) into SBC. Specifically, 10 g of SBC and varying amounts of ZnCl_2_ or FeCl_3_ (2.5, 5.0, 10.0, and 20.0 g) were mixed in a beaker containing 150 mL of ultrapure water. The mixture was stirred at 85 °C for 2 h using a magnetic stirrer and then dried in an oven at 105 °C for 24 h under ambient atmosphere to remove excess water. The dried mixture was placed in a quartz tube and subjected to pyrolysis under nitrogen flow at a heating rate of 5 °C min^−1^, maintaining a temperature of 550 °C for 2 h. The resulting product was washed with 3 M HCl for 30 min at approximately 90 °C to remove residual impurities, followed by thorough washing with ultrapure water until a neutral pH was achieved. The final product was dried in an oven at 105 °C for 12 h and sieved through a 100-mesh sieve to obtain ZnCl_2_ or FeCl_3_ modified biochar (designated as XZn-YSBC or XFe-YSBC, where X/Y represents the ratio of Zn or Fe to SBC content: 1/4, 1/2, 1/1, or 2/1).

#### 3.2.2. Bimetallic Modified Biochar

Bimetallic modified biochar was prepared by mixing SBC with both ZnCl_2_ and FeCl_3_. A mixture containing 10 g of SBC, 10 g of ZnCl_2_, and varying amounts of FeCl_3_ (2.5, 5.0, 10.0, and 20.0 g) was stirred in a beaker with 150 mL of ultrapure water at 85 °C for 2 h. The mixture was then dried in an oven at 105 °C for 24 h under an ambient atmosphere to remove water and activate the SBC. The activated mixture was subjected to pyrolysis in a quartz tube under nitrogen flow, with the heating rate and temperature parameters identical to those for monometallic biochar. The pyrolyzed product was washed with 3 M HCl, heated to approximately 90 °C, and rinsed with ultrapure water until a neutral pH was achieved. The final product was dried at 105 °C for 12 h and sieved through a 100-mesh sieve to obtain bimetallic modified biochar (designated as XZn-YFe-1SBC, where X/Y represents the Zn to Fe ratio: 4/1, 2/1, 1/1, or 1/2).

### 3.3. Material Characterization

The synthesized biochar materials were characterized using a range of analytical techniques to assess their physical and chemical properties. Fourier transform infrared spectroscopy (FTIR, NiCoLET- iS10 model, Perkin Elmer Inc., Waltham, MA, USA) was employed to identify functional groups, with the following operating conditions: number of scans (50), resolution (4 cm^−1^), spectral range (4000 to 500 cm^−1^), and acquisition time (2.47 s). The samples were prepared by the standard KBr pellets method. Scanning electron microscopy (SEM, Hitachi High-Tech Corporation, Tokyo, Japan) coupled with energy-dispersive X-ray spectroscopy (EDS, S-4700 model, Hitachi High-Tech Science Corporation, Tokyo, Japan) provided insights into surface morphology and elemental composition. X-ray photoelectron spectroscopy (XPS, Nexsa G2 model, Thermo Fisher Scientific Inc., Waltham, MA, USA) was utilized to examine the chemical states and crystal structures, with calibration performed by the ISO 15472 method under an excitation wavelength of 290 nm and a scanned area of 5 mm. Nitrogen adsorption/desorption isotherm analysis (BET, ASAP 2020 model, Micromeritics Inc., Norcross, GA, USA) was conducted to determine specific surface area and pore structure. Thermogravimetric analysis (TGA, ZCT-1 model, TA Instruments Inc., New Castle, DE, USA) and elemental analysis (EA, MICRO cube model, Elementar Analysensysteme GmbH Inc., Langenselbold, Germany) were performed to evaluate the thermal stability and elemental composition of the biochar materials.

### 3.4. Material Performance Valuation

The adsorption performance of the biochar materials was assessed through batch experiments targeting As(III) and oxytetracycline (OTC) in aqueous solutions. The concentration of As(III) was measured using inductively coupled plasma mass spectrometry (ICP-MS, 4100 MP-AES model, Thermo Fisher Scientific Inc., SA, USA). The instrument has a detection limit of 10 ppb. To ensure data reliability, all experiments were conducted in triplicate, yielding a standard deviation of 20 ppb. Calibration with standard solutions was performed regularly to maintain accuracy.

For OTC, the adsorption capacity was evaluated using a UV-Vis spectrophotometer (U-3900 model, Hitachi High-Tech Science Corporation, Tokyo, Japan). A standard 1 mg L^−1^ OTC solution was prepared, and the maximum absorption wavelength (272 nm) was determined through multiple wavelength scans. The concentration changes in OTC after adsorption by modified biochar were calculated based on the absorbance values, providing insights into the material’s performance in removing OTC from aqueous solutions.

### 3.5. Isothermal Adsorption Model

#### 3.5.1. Langmuir Isotherm Model

The Langmuir isotherm model is widely used to describe the adsorption of solute species onto solid adsorbents. It assumes monolayer adsorption on a surface with a finite number of identical sites. The theoretical derivation leads to an equation that establishes a relationship between the surface’s active sites undergoing adsorption and the equilibrium concentration. The Langmuir model is expressed as [64]:

(1) $$Q=(\frac{ab\times C_{e}}{1+aC_{e}})$$

Taking the reciprocal of both sides results in:

(2) $$\frac{1}{Q}=\frac{1}{b}+\frac{1}{ab\times C_{e}}$$

Here, *C_e_* is the equilibrium concentration of the adsorbate in solution, and *Q* is the amount of adsorbate adsorbed per unit mass of adsorbent. The constants *a* and *b* represent the adsorption energy and capacity, respectively. By plotting 1/*Q* against 1/*C_e_*, the values of *a* and *b* can be determined from the slope and intercept.

#### 3.5.2. Freundlich Isotherm Model

The Freundlich isotherm is an empirical model describing the relationship between the concentration of the adsorbate on the adsorbent surface and its concentration in solution. It is expressed as [65]:

(3) $$Q=K{C_{e}}^{\frac{1}{n}}$$

Taking the natural logarithm of both sides yields:

(4) $$\ln Q=\ln K+(\frac{1}{n\;(\ln C_{e})})$$

Here, *K* and *n* are the Freundlich constants related to adsorption capacity and intensity, respectively. By plotting ln*Q* against *C_e_*, *K*, and *n* can be obtained from the intercept and slope, respectively.

#### 3.5.3. Temkin Isotherm Model

The Temkin isotherm model accounts for adsorbent-adsorbate interactions by assuming that the heat of adsorption decreases linearly with coverage rather than logarithmically. It is derived based on a uniform distribution of binding energies. The model is represented by the equations [66]:

(5) $${q_{T}=q}_{e}=\frac{\mathrm{RT}}{b}\ln\left(A_{T}\;C_{e}\right)$$

(6) $$q_{e}=\left(\frac{\mathrm{RT}}{b}\right)\ln A_{T}+\left(\frac{\mathrm{RT}}{b}\right)\ln C_{e}$$

In these equations, q_T_ represents the adsorption capacity predicted by the Temkin model (mg g^−1^), q_e_ is the equilibrium adsorption capacity (mg g^−1^), R is the universal gas constant (8.314 J mol^−1^ K^−1^), T is the absolute temperature (K), b is the Temkin constant, A_T_ is the Temkin equilibrium binding constant (L g^−1^), and C_e_ is the equilibrium concentration (ppm).

### 3.6. Adsorption Kinetics Models

#### 3.6.1. Pseudo-First-Order Kinetics

The pseudo-first-order kinetic model assumes that the adsorption rate is proportional to the concentration of unadsorbed species. The equation is given as:

(7) $$\frac{dq_{t}}{dt}=k_{1}(q_{e}-q_{t})$$

Here, *q_t_* is the amount adsorbed at time *t* (mg g^−1^), *k*_1_ is the pseudo-first-order rate constant (min^−1^), and *q_e_* is the adsorption capacity at equilibrium. Integrating this equation with the initial condition *q_t_* = 0 at *t* = 0 yields [67]:

(8) $$\log(q_{e}-q_{t})=\log q_{e}-\frac{k_{1}}{2.303}t$$

#### 3.6.2. Pseudo-Second-Order Kinetics

The pseudo-second-order kinetic model assumes that the adsorption rate depends on the square of the concentration of unadsorbed species. It is described as:

(9) $$\frac{dq_{t}}{dt}=k_{2}{\left(q_{e}-q_{t}\right)}^{2}$$

Integrating this equation with the condition *q_t_* = 0 at *t* = 0 gives [68]:

(10) $$\frac{t}{q_{t}}=\frac{1}{k_{2}{q_{e}}^{2}}+\frac{1}{q_{e}}t$$

Here, *k*_2_ is the pseudo-second-order rate constant (g mg^−1^ min^−1^).

#### 3.6.3. Intra-Particle Diffusion Model

The intra-particle diffusion model evaluates the rate of adsorption based on the square root of time. It is expressed as:

*q_t_* = *k_i_*t^1/2^ + *C* (11)

Here, *k_i_* (mg g^−1^ min^−1/2^) is the rate constant for intra-particle diffusion, and *C* is a constant related to boundary layer thickness [69,70].

### 3.7. Thermodynamic Model

Thermodynamics primarily utilizes the relationships between Gibbs free energy (ΔG), entropy (ΔS), and enthalpy (ΔH) to analyze the reaction mechanisms of pollutants in systems undergoing temperature changes [71]. The adsorption process is classified as spontaneous when ΔG is less than 0 kJ mol^−1^ and as non-spontaneous when ΔG equals 0 kJ mol^−1^. Conversely, if ΔG is greater than 0 kJ mol^−1^, the adsorption process is again considered spontaneous. The ΔH value further elucidates the nature of the adsorption process. When ΔH is less than 0 kJ mol^−1^, the process is exothermic, indicating that adsorption is more effective at lower temperatures. In contrast, a ΔH greater than 0 kJ mol^−1^ signifies an endothermic process, where the adsorption capacity decreases as the temperature increases. Additionally, the magnitude of ΔH provides insight into the type of adsorption: values between 0 and 80 kJ mol^−1^ indicate physical adsorption, while values between 80 and 400 kJ mol^−1^ suggest chemical adsorption [72,73]. The thermodynamic equations used are as follows:

(12) $$K_{d}=q_{e}/C_{e}$$

(13) $$\Delta G=\;-\mathrm{RT}\times \ln(K_{d})$$

(14) $$\ln(K_{d})=\frac{\Delta S}{R}+\frac{-\Delta H}{\mathrm{RT}}$$

where, K_d_ is the reaction equilibrium constant (L mg^−1^), C_e_ is the concentration of adsorption reaction equilibrium (mg L^−1^), q_e_ is the adsorption capacity at adsorption equilibrium (mg g^−1^).

### 3.8. Arrhenius Model

The Arrhenius equation relates the reaction rate constant (*k*) to temperature, providing insights into activation energy (E_a_) as follows:

(15) $$k=A\times e^{-\frac{E_{a}}{\mathrm{RT}}}$$

Taking the natural logarithm yields:

(16) $$\ln(k)=\ln(A)\;-\;\left(\frac{E_{a}}{\mathrm{RT}}\right)$$

Here, A is the pre-exponential factor, and E_a_ is the activation energy (kJ mol^−1^) [74].

## 4. Conclusions

The SEM-EDS analysis revealed that the modification of biochar with ZnCl_2_ and FeCl_3_ significantly enhanced pore formation and facilitated the loading of Zn and Fe particles onto the surface of 1Zn-1Fe-1SBC. Following the adsorption of As(III) by 1Zn-1Fe-1SBC, the presence of As elements was confirmed on the material’s surface. TGA analysis demonstrated that 1Zn-1Fe-1SBC exhibited high thermal stability at elevated temperatures. BET analysis indicated that the specific surface area and pore volume of the biochar increased markedly after ZnCl_2_ and FeCl_3_ modification, from 16.8 m^2^ g^−1^ to 1136.8 m^2^ g^−1^ and from 0.02 cm^3^ g^−1^ to 0.55 cm^3^ g^−1^, respectively. However, both the specific surface area and pore volume decreased significantly to 945.7 m^2^ g^−1^ and 0.43 cm^3^ g^−1^, respectively, after the adsorption of As(III) and OTC by 1Zn-1Fe-1SBC. FTIR analysis identified an As-OH bending vibration at 852 cm^−1^ following the adsorption of As(III), confirming the interaction between As(III) and the material. XPS analysis revealed that the ratios of oxygen-containing functional groups, such as C-O and C=O, as well as π-π interactions, were significantly higher in the modified biochar compared to the unmodified biochar. This enhancement in functional groups and π-π electron interactions facilitated the effective adsorption of As(III) and OTC by 1Zn-1Fe-1SBC. These findings underscore the effectiveness of ZnCl_2_ and FeCl_3_ modifications in improving the adsorption performance and thermal stability of biochar, making 1Zn-1Fe-1SBC a promising material for environmental remediation applications.

## Figures and Tables

**Table 1:** Specific surface area and pore structure of various biochars.

Materials	Surface Area (m^2^ g^−1^)	Average Pore Volume (cm^3^ g^−1^)	Pore Size (nm)
SBC	16.8	0.02	4.65
1Zn-1SBC	1314.5	0.64	1.92
1Fe-1SBC	97.3	0.31	3.28
1Zn-1Fe-1SBC	1136.8	0.55	1.98
1Zn-1Fe-1SBC-As-OTC	945.7	0.43	3.05
1Zn-1Fe-1SBC-5 cycles	989.5	0.45	2.31

**Table 2:** EDS elemental analysis results.

	SCB		SBC		1Zn-1Fe-1SBC		1Zn-1Fe-1SBC-As-OTC	
	W (%)	A (%)	W (%)	A (%)	W (%)	A (%)	W (%)	A (%)
C (K)	55.84	62.78	86.75	89.97	80.37	86.57	65.08	78.30
O (K)	44.04	37.14	12.38	9.74	15.17	12.26	19.01	17.17
Si (K)	0.12	0.08	0.87	0.39	0.26	0.12	1.52	0.78
Zn (L)	-	-	-	-	0.09	0.02	0.45	0.10
Fe (K)	-	-	-	-	4.11	1.03	13.86	3.63
As (L)	-	-	-	-	-	-	0.08	0.02
N (K)	-	-	-	-	-	-	0.40	0.01
Total	100	100	100	100	100	100	100	100

**Table 3:** Parameters for the analysis of isothermal adsorption model of biochar.

Model	Langmuir			Freundlich			Temkin		
	K_L_ (L mg^−1^)	Q_m_ (mg g^−1^)	R^2^	K_F_ (L g^−1^)	n	R^2^	K_T_ (kJ mol^−1^)	A_T_ (m^3^ mole^−1^)	R^2^
1Zn-1Fe- 1SBC-As	0.15	34.72	0.999	4.22	1.20	0.996	610.06	4.08	0.929
1Zn-1Fe- 1SBC-OTC	0.03	172.41	0.999	5.26	1.16	0.997	141.92	0.99	0.926

**Table 4:** Parameters for analysis of biochar kinetic model.

Model		Pseudo-First-Order			Pseudo-Second-Order		
		k_1_	q_e_	R^2^	k_2_	q_e_	R^2^
1Zn-1Fe- 1SBC-AS	1 ppm	0.125	1.151	0.994	0.317	1.276	0.980
	2 ppm	0.083	2.154	0.986	0.135	2.423	0.968
	4 ppm	0.061	3.626	0.948	0.090	4.114	0.976
	8 ppm	0.051	5.577	0.886	0.078	6.325	0.986
	16 ppm	0.043	9.442	0.845	0.054	10.132	0.989
1Zn-1Fe- 1SBC-OTC	1 ppm	0.210	4.65	0.992	0.154	5.13	0.994
	2 ppm	0.142	8.75	0.973	0.059	10.1	0.990
	4 ppm	0.078	13.1	0.936	0.033	17.5	0.989
	8 ppm	0.054	23.3	0.894	0.019	27.5	0.987
	16 ppm	0.067	45.2	0.973	0.007	50.5	0.970

**Table 5:** Parameters of biochar particle diffusion analysis.

Model	First Stage		Second Stage		Third Stage	
	K_1_ (mg/g min^0.5^)	R^2^	K_2_ (mg/g min^0.5^)	R^2^	K_3_ (mg/g min^0.5^)	R^2^
1Zn-1Fe- 1SBC-AS	0.922	0.997	0.411	0.993	0.116	0.997
1Zn-1Fe- 1SBC-OTC	3.012	0.998	1.390	0.999	0.246	0.996

**Table 6:** Parameters of thermodynamic models for the adsorption of As(III) and OTC by various biochars.

Material	Temperature (K)	ΔG (kJ mol^−1^)	ΔH (kJ mol^−1^)	ΔS (J mol^−1^ K^−1^)
1Zn-1Fe- 1SBC-As	288	−7.026	82.60	309.61
	298	−9.167		
	308	−12.26		
	318	−16.40		
1Zn-1Fe- 1SBC-OTC	288	−10.49	84.45	329.60
	298	−13.47		
	308	−17.64		
	318	−20.07		

**Table 7:** Arrhenius model analysis of biochar.

Material	Temperature (K)	k_1_	E_a_ (kJ mol^−1^)	A (s^−1^)	R^2^
1Zn-1Fe- 1SBC-As	288	0.020	21.82	32.92	0.954
	298	0.024			
	308	0.029			
	318	0.041			
1Zn-1Fe- 1SBC-OTC	288	0.021	26.07	754.3	0.985
	298	0.027			
	308	0.042			
	318	0.052			

## Data Availability

The data presented in this study are available in this article.

## Supplementary Materials

The following supporting information can be downloaded at: https://www.mdpi.com/article/10.3390/molecules30030572/s1, Figure S1: SEM and EDS mapping of 1Zn-1Fe-1SBC. (a) SEM image of 1Zn-1Fe-1SBC, (b) C, (c) O, (d) Si, (e) Fe, and (f) Zn.; Figure S2: SEM and EDS mapping of 1Zn-1Fe-1SBC-As-OTC. (a) SEM image of 1Zn-1Fe-1SBC-As-OTC, (b) C, (c) O, (d) Si, (e) Fe, (f) Zn, (g) As, and (h) N.; Figure S3. SEM and EDS mapping of SCB. (a) SEM image of SCB, (b) C, (c) O, and (d) Si.; Figure S4. SEM and EDS mapping of SBC. (a) SEM image of SBC, (b) C, (c) O, and (d) Si.; Figure S5. Effect of different pH values on As(III) adsorption capacity and efficiency. (a) SBC, (b) 1Zn-1SBC, (c)1Fe-1SBC, and (d) 1Zn-1Fe-SBC.; Figure S6. Effect of pH on the isoelectric potential of materials.; Figure S7. Effect of different pH values on OTC adsorption capacity and efficiency. (a) SBC (b) 1Zn-1SBC (c)1Fe-1SBC (d) 1Zn-1Fe-SBC.; Figure S8. Isothermal adsorption mode analysis of As(III) adsorption results. (a) As(III), and (b) OTC.
