# Supplementary material for: Bimetallic Zinc-Iron-Modified Sugarcane Bagasse Biochar for Simultaneous Adsorption of Arsenic and Oxytetracycline from Wastewater
Source: Molecules. 2025 Jan 27;30(3):572. doi: 10.3390/molecules30030572 (PMC11820934; doi:10.3390/molecules30030572)
Supplement: Supplementary file 1 [file molecules-30-00572-s001.zip › molecules-3429880-supplementary.pdf]

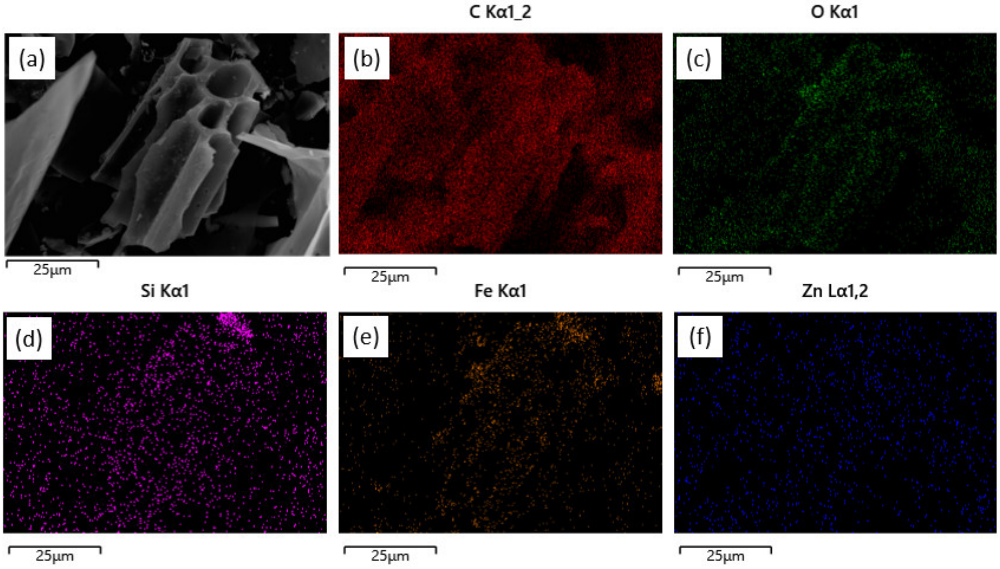

Figure S1. SEM and EDS mapping of 1Zn-1Fe-1SBC. (a) SEM image of 1Zn-1Fe-1SBC, (b) C, (c) O, (d) Si, (e) Fe, and (f) Zn.

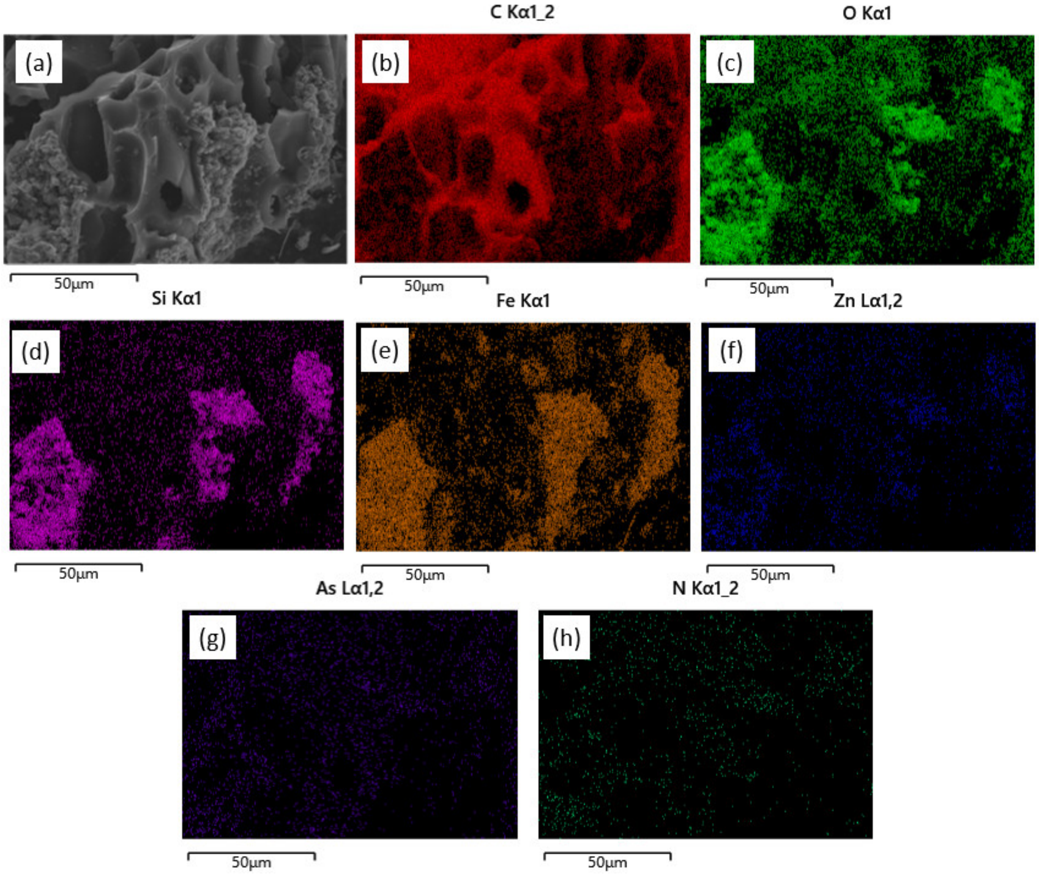

Figure S2. SEM and EDS mapping of 1Zn-1Fe-1SBC-As-OTC. (a) SEM image of 1Zn-1Fe-1SBC-As-OTC, (b) C, (c) O, (d) Si, (e) Fe, (f) Zn, (g) As, and (h) N.

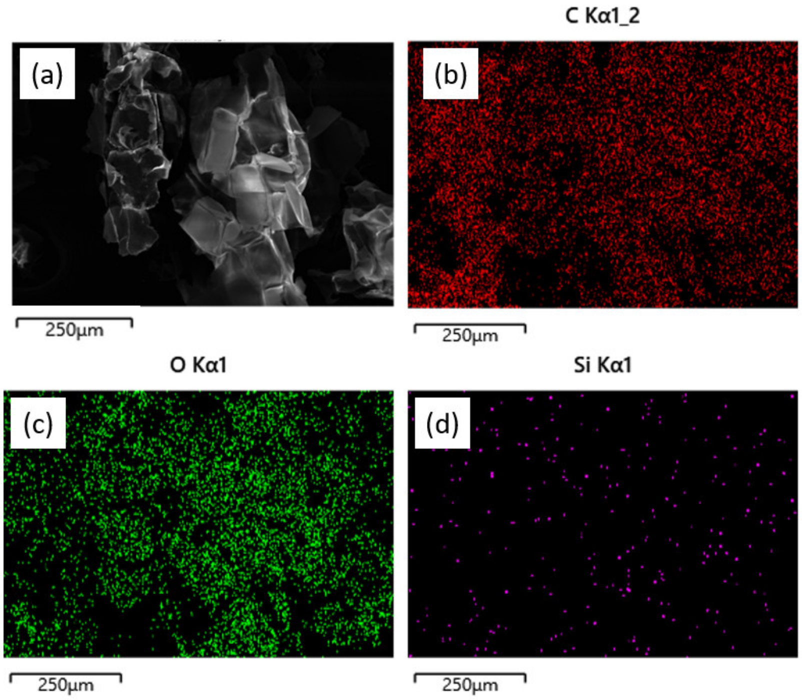

Figure S3. SEM and EDS mapping of SCB. (a) SEM image of SCB, (b) C, (c) O, and (d) Si.

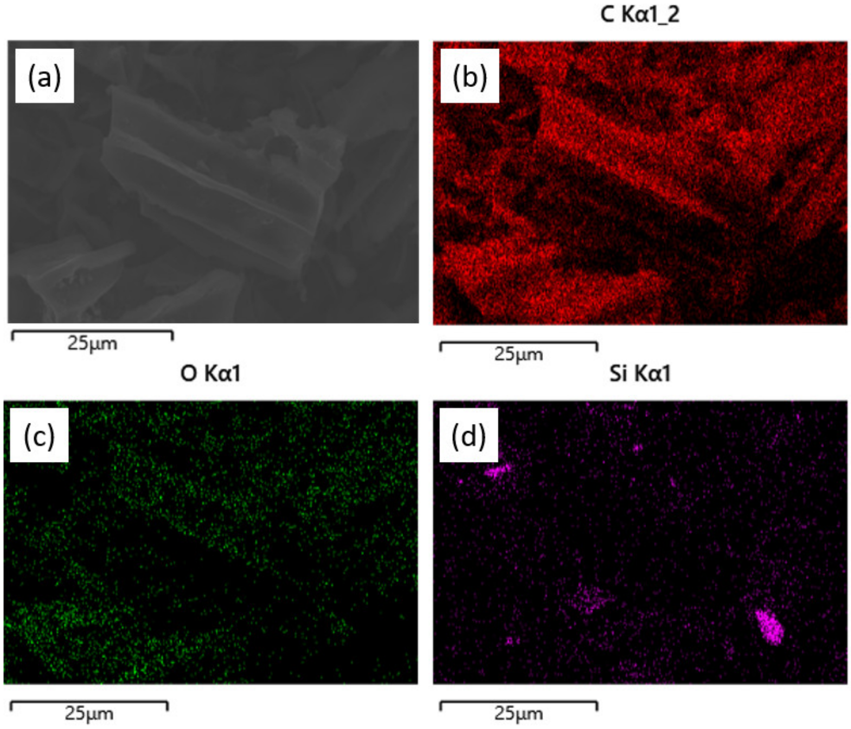

Figure S4. SEM and EDS mapping of SBC. (a) SEM image of SBC, (b) C, (c) O, and (d) Si.

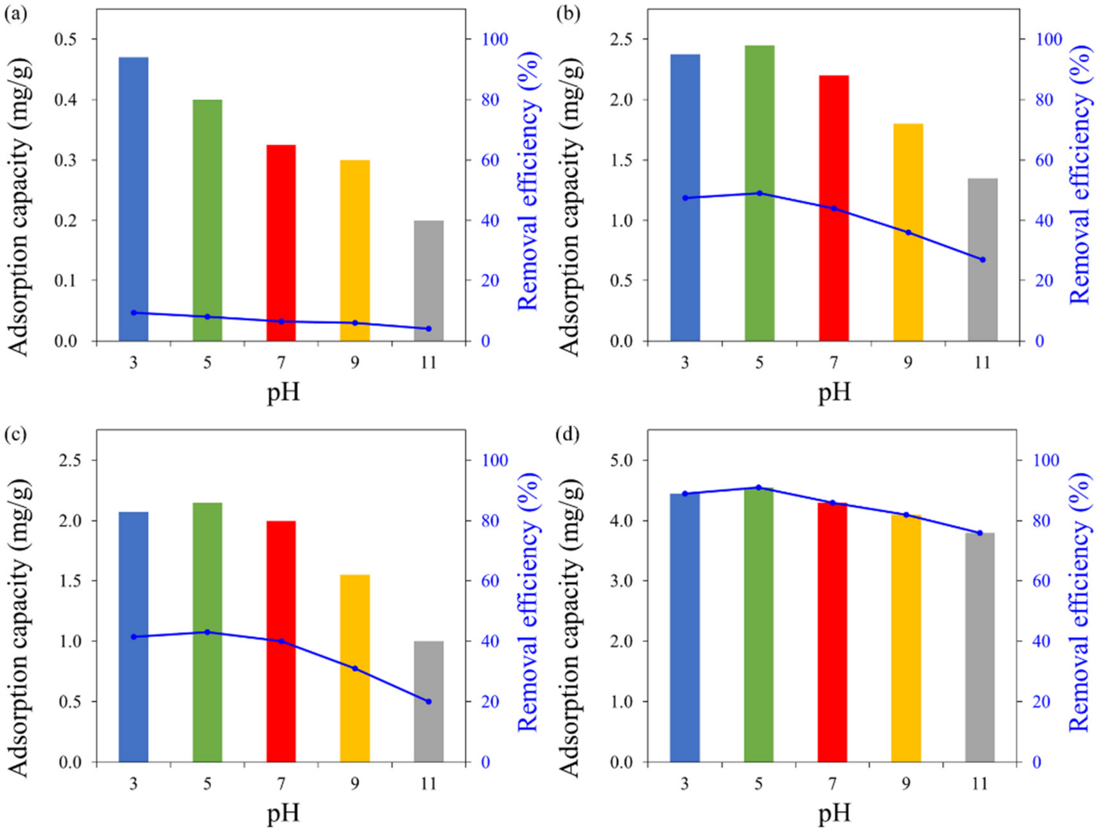

Figure S5. Effect of different pH values on As(III) adsorption capacity and efficiency. (a) SBC, (b) 1Zn-1SBC, (c)1Fe-1SBC, and (d) 1Zn-1Fe-SBC.

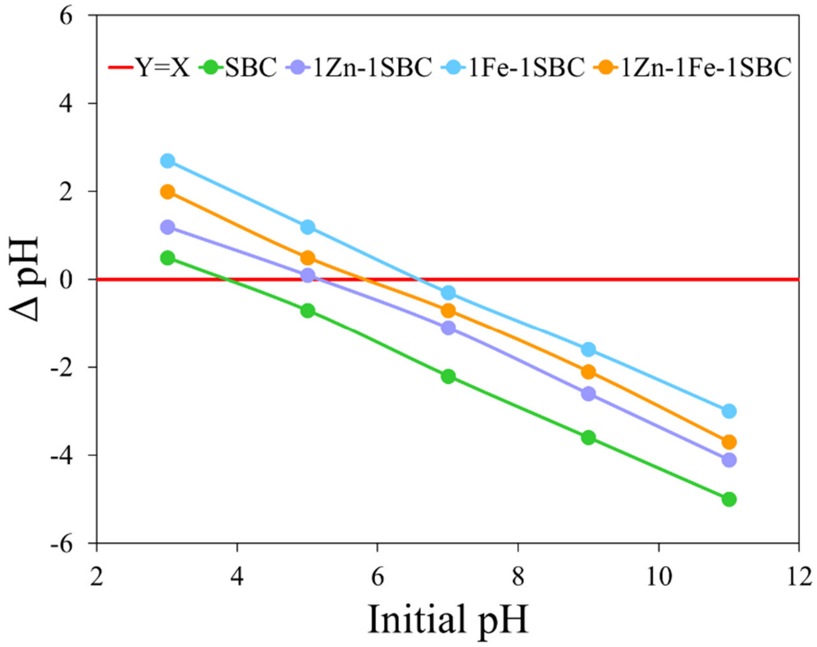

Figure S6. Effect of pH on the isoelectric potential of materials.

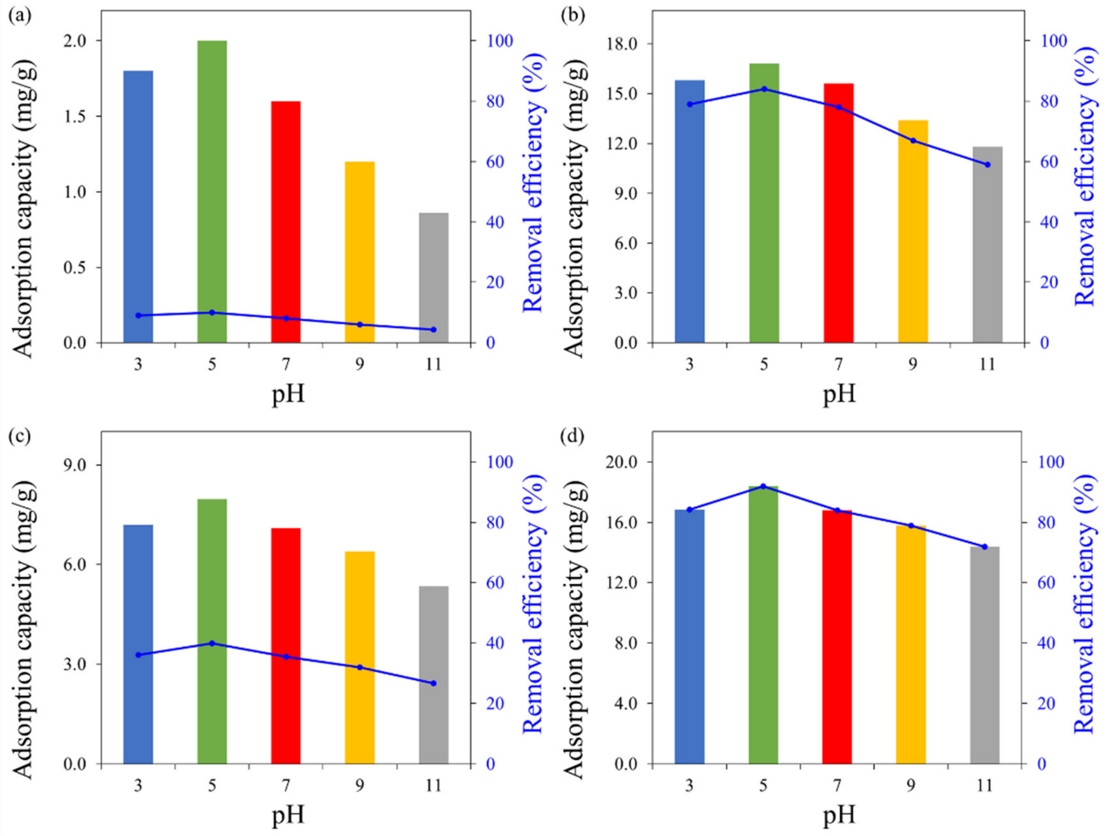

Figure S7. Effect of different pH values on OTC adsorption capacity and efficiency. (a) SBC (b) 1Zn-1SBC (c)1Fe-1SBC (d) 1Zn-1Fe-SBC.

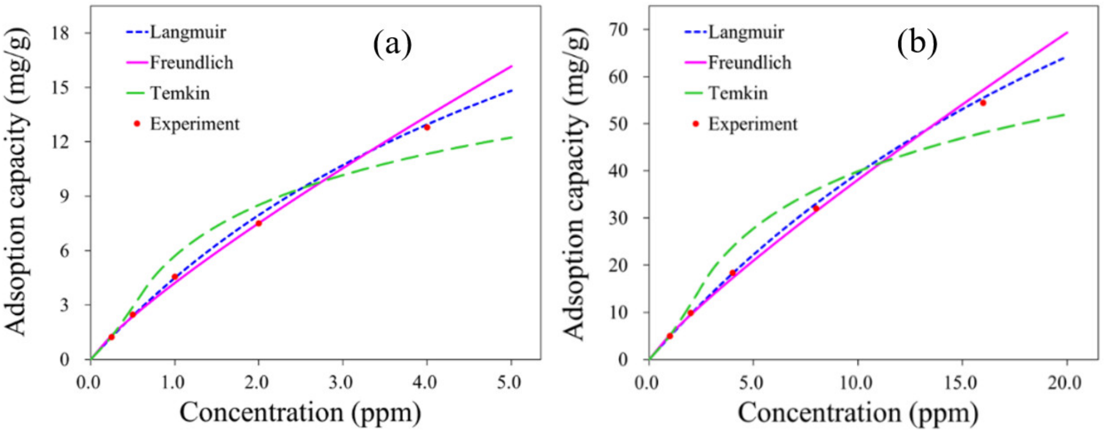

Figure S8. Isothermal adsorption mode analysis of As(III) adsorption results. (a) As(III), and (b) OTC.
